# Physical activity partially mediates the association between health literacy and mild cognitive impairment in older adults: cross-sectional evidence from Switzerland

**DOI:** 10.1093/eurpub/ckae209

**Published:** 2025-01-03

**Authors:** Clément Meier, Maud Wieczorek, Damaris Aschwanden, Andreas Ihle, Matthias Kliegel, Jürgen Maurer

**Affiliations:** Faculty of Business and Economics (HEC), University of Lausanne, Lausanne, Switzerland; Swiss Centre of Expertise in the Social Sciences (FORS), University of Lausanne, Lausanne, Switzerland; Swiss Center of Expertise in Life Course Research LIVES, Lausanne and Geneva, Switzerland; Faculty of Business and Economics (HEC), University of Lausanne, Lausanne, Switzerland; Swiss Center of Expertise in Life Course Research LIVES, Lausanne and Geneva, Switzerland; Swiss Center of Expertise in Life Course Research LIVES, Lausanne and Geneva, Switzerland; Centre for the Interdisciplinary Study of Gerontology and Vulnerability, University of Geneva, Geneva, Switzerland; Swiss Center of Expertise in Life Course Research LIVES, Lausanne and Geneva, Switzerland; Centre for the Interdisciplinary Study of Gerontology and Vulnerability, University of Geneva, Geneva, Switzerland; Department of Psychology, University of Geneva, Geneva, Switzerland; Swiss Center of Expertise in Life Course Research LIVES, Lausanne and Geneva, Switzerland; Centre for the Interdisciplinary Study of Gerontology and Vulnerability, University of Geneva, Geneva, Switzerland; Faculty of Business and Economics (HEC), University of Lausanne, Lausanne, Switzerland; Swiss Center of Expertise in Life Course Research LIVES, Lausanne and Geneva, Switzerland

## Abstract

Individuals’ health literacy (HL) is positively associated with healthy behaviors and global cognitive functioning. Current evidence also suggests that physical activity may prevent or delay cognitive decline and dementia. This study examines the potential mediating role of physical activity in the association between HL and cognition in a population-based sample of adults aged 58+ in Switzerland. We used data from 1645 respondents to Wave 8 (2019/2020) of the Survey on Health, Ageing, and Retirement in Europe in Switzerland. HL was assessed using the HLS-EU-Q16 questionnaire. Mild cognitive impairment (MCI) was defined as a 1.5 SD below the mean of age- and education-specific global cognition score. The frequency of moderate and vigorous physical activity was self-reported. The associations were assessed using probit regression models, controlling for social, health, and regional characteristics. Structural equation modeling was used to test the mediation hypothesis. Higher HL was associated with a higher likelihood of being engaged in moderate (*P* < .001) and vigorous (*P* < .01) physical activity and with a lower likelihood of having MCI (*P* < .05). In addition, both moderate (*P* < .05) and vigorous (*P* < .01) physical activity were associated with a lower probability of having MCI. Mediation analysis indicated that the association between HL and MCI was partially mediated by both moderate (12.9%) and vigorous (6.7%) physical activity. Given that physical activity may partially mediate the association between HL and MCI, improving HL in older adults could potentially foster engagement in physical activity, which could, in turn, act as a protective factor against MCI.

## Introduction

The interplay between health literacy (HL) and cognitive outcomes in older adults has gathered significant attention due to the increasing prevalence of cognitive impairments and dementia worldwide [1]. Health literacy, defined as the ability to access, understand, appraise, and apply health information, is a critical determinant of health outcomes and behaviors [2]. Notably, higher levels of HL are associated with healthier lifestyle choices and better cognitive functioning, which are pivotal for aging populations [3]. More precisely, extensive research suggests that higher levels of HL are associated with better performance on cognitive tests that assess global cognitive function [4], as well as specific domains such as immediate recall, delayed recall, and verbal fluency [5]. Furthermore, this association remains robust even in tests that are independent of reading ability or education level, and after accounting for individuals’ health conditions, indicating that lower cognitive scores in individuals with limited HL reflect differences in cognitive functioning [6]. Other studies provided comprehensive evidence that individuals’ HL is strongly associated with global cognitive functioning and healthy behaviors, corroborating the idea that better HL can lead to healthier lifestyle choices [7]. Emerging evidence highlights that individuals with higher HL are more likely to engage in physical activity [8], which reinforces the importance of HL in promoting physical activity and, consequently, better health outcomes [9].

In addition, physical activity is a potential modifiable factor that can mitigate the risk of cognitive decline and dementia [10]. Mechanistically, exercise contributes to cognitive health by providing physiological benefits such as improved cerebrovascular health and increased neuroplasticity, which not only help maintain cognitive functions in older adults but also improve cerebrovascular conditions and address risk factors such as hypertension and diabetes, thereby contributing to overall brain health [11]. Physical activity is also associated positively with the quality of life across both frail and non-frail individuals [12], healthy aging [13], decreased depression levels [14], a protective effect against Alzheimer’s disease [15], and prolonged survival among Alzheimer’s disease patients [16]. The protective effect of physical activity and the risk of cognitive decline and dementia could even be achieved with moderate levels of physical activity [17]. More precisely, the results of a systematic review indicated that multicomponent exercise training, which combines aerobic and resistance elements, was found to be more effective in enhancing cognitive performance [18]. Therefore, understanding the pathways through which HL is associated with physical activity and, subsequently, cognitive health is essential for developing targeted interventions aimed at promoting healthy aging. However, while the links between HL, physical activity, and cognitive health are established, the specific mediating role of physical activity in the relationship between HL and cognitive function has not been thoroughly investigated.

**Figure 1.**
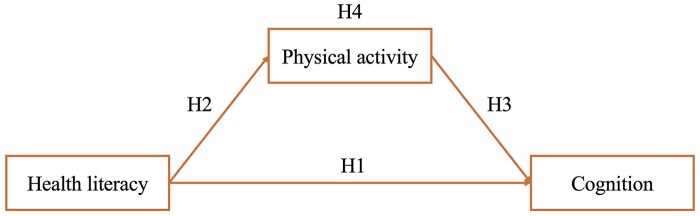
Hypotheses on the potential mediating role of physical activity in the association between health literacy and cognition. H1: Individuals’ health literacy level is negatively associated with mild cognitive impairment. H2: Individuals’ health literacy level is positively associated with physical activity. H3: Individuals’ physical activity is negatively associated with mild cognitive impairment. H4: The association between individuals’ health literacy level and mild cognitive impairment is partially mediated by physical activity.

This study seeks to fill this critical gap by examining the hypothesis that physical activity acts as a mediator in the relationship between HL and cognition among older adults in Switzerland (Fig. 1). By leveraging data from a robust population-based survey, we aim to elucidate the extent to which engaging in moderate and vigorous physical activities can mediate the association between HL and the presence of mild cognitive impairment (MCI). The findings of this research could inform the design of public health interventions that enhance HL and encourage physical activity to protect cognitive health, ultimately contributing to the broader goals of healthy aging programs. Given the aging demographic trends and the considerable burden of cognitive impairments on individuals, families, and healthcare systems, this study could have significant implications for public health policy and practice.

## Methods

### Study design and participants

This study examined data from individuals who responded to a specific paper-and-pencil questionnaire for Switzerland only, which was added to the main face-to-face interview of the 8th Wave of the Survey on Health, Ageing, and Retirement in Europe (SHARE), conducted from October 2019 to March 2020 [19, 20]. SHARE is an extensive longitudinal study that gathers detailed information on health, socio-economic status, and social and family networks from individuals aged 50 and older, along with their partners, across 27 European countries and Israel. The study obtained ethical approval number 66/14 from the ethics committee of the canton of Vaud, Switzerland, in March 2014. During Wave 8, 2005 Swiss respondents and their partners answered the in-person interviews, and most of them, 1891 (94.3%), also filled out the additional national paper-and-pencil questionnaire. The analysis for this specific study focused on respondents aged 58 and above, excluding those between 50 and 57 years, due to the Swiss cohort sample not having been refreshed since 2011. Furthermore, it excluded respondents who did not answer all questions relevant to the analysis done in this article. As a result, the study’s effective sample size was narrowed down to 1645 participants.

### Outcome variable

#### Mild cognitive impairment

Cognitive functioning was evaluated during the computer-assisted personal interviews. The present study focused on three essential cognitive abilities: immediate memory, delayed memory, and verbal fluency, which were all objectively tested. The modified Rey’s Auditory Verbal Learning Test [21] was utilized to assess both immediate and delayed memory capabilities through immediate and delayed recall tasks involving a list of 10 words (Appendix S1). Verbal fluency was determined by counting the number of unique animals named by a participant within a minute (Appendix S2). Higher scores in these tests indicated better cognitive performance. Cognitive scores were adjusted for age and educational background to normalize for individual variations. Age was categorized into three groups: 58–64, 65–74, and 75+ years. Education levels were defined as low, medium, and high according to the 2017 International Standard Classification of Education (ISCED) [22]. The average and standard deviation for each cognitive skill were calculated across the nine demographic groups created by intersecting age and educational categories. Each cognitive ability’s raw score was then converted to a *z*-score by subtracting the category-specific mean and dividing by its standard deviation. A composite cognitive score was calculated by averaging the *z*-scores across the three abilities, with higher *z*-scores indicating better cognitive function. Finally, a binary variable was established to identify MCI, defined as scoring 1.5 SDs below the age- and education-adjusted mean on the global cognition score [23].

### Exposure

#### Health literacy

The specific questionnaire for Switzerland included the 16-item short scale of the European Health Literacy Survey, developed by the HLS-EU consortium (HLS-EU-Q16) [24]. Respondents assess their ability to handle specific health-related tasks or situations using a 4-point Likert scale that ranges from “very easy,” “fairly easy,” “fairly difficult,” to “very difficult” (Appendix S3). In line with methods recommended by HLS-EU researchers, responses of “fairly difficult” and “very difficult” are coded as “0,” while “very easy” and “fairly easy” are coded as “1.” Any missing responses are also recorded as “0.” To calculate the final HL score, only those respondents with no more than two missing item responses are included. Of the total, 134 respondents (7%) had one or two incomplete responses among the 16 items. The final score is then normalized by dividing it by the standard deviation [3], yielding a score between 0 and 5.3. Higher scores indicated better HL.

### Potential mediator

#### Physical activity

In the main questionnaire from SHARE Wave 8, respondents were asked how often they engaged in vigorous physical activities such as sports, heavy housework, or a job that involved physical labor. Additionally, they were asked about their frequency of participation in activities requiring a moderate level of energy, such as gardening, cleaning the car, or walking. Respondents assessed their physical activities using a 4-point Likert scale that ranges from “More than once a week,” “Once a week,” “One to three times a month,” to “Hardly ever or never” (Appendix S4). Engagement in moderate physical activity was defined as participating in the listed moderate activities at least once a week. Similarly, engagement in vigorous physical activity required participation in the listed vigorous activities at least once a week. Consequently, two binary variables were created and used for the analysis, with a value of 1 assigned to respondents answering “More than once a week” or “Once a week,” and a value of 0 for those selecting “One to three times a month,” “Hardly ever or never.”

### Covariates

The analysis was adjusted for several factors, including sex (0 = male, 1 = female), age categories (58–64 years, 65–74 years, 75+ years), and levels of education according to the ISCED (low = 0–2, medium = 3–4, high = 5–6) [22]. Additionally, adjustments were made for the presence of a partner (0 = has a partner, 1 = no partner) and respondents’ self-assessed financial situation, which was determined by their ability to make ends meet, with responses categorized as 1 = “easily,” 2 = “fairly easily,” and 3 = “with difficulty.” The language of the questionnaire responses was also used to identify the linguistic region of Switzerland the respondent was from (Swiss-German, French, or Swiss-Italian). Self-reported information about the respondents’ living environment was captured by whether they resided in an urban or rural setting (0 = urban, 1 = rural). Health status was evaluated through two additional variables: one assessing self-rated health (1 = “poor/fair,” 2 = “good,” 3 = “very good/excellent”) and another assessing any difficulties the respondents faced with daily living activities (ADL; 0 = no difficulties, 1 = has difficulties), thus providing insights into functional health [25, 26].

### Statistical analysis

The demographic characteristics of the analytical sample were detailed using number counts and their respective percentages. The partial associations between the HL score, moderate and vigorous physical activity, and MCI were analyzed using separate probit regression models. The analysis adjusted for variables including sex, age groups, education levels, partnership status, subjective financial status, living area, linguistic region, self-rated health, and ADL. The results were presented as average marginal effects (AME) with their respective standard errors (SE). Given the possibility of unobserved dependencies due to the inclusion of respondents and their partners in the SHARE survey, SE were clustered at the household level within the multivariable models. To explore the respective mediating role of moderate and vigorous physical activity in the association between HL score and MCI, two structural equation modeling (SEM) analyses were done. The criteria used to ensure a good model fit included the Tucker–Lewis index (TLI > 0.95), the comparative fit index (CFI > 0.95), and the root mean square error of approximation (RMSEA < 0.06) [27]. SEM analyses also controlled for the same covariates mentioned above. The statistical analyses were performed using STATA/SE 18.0 (STATA Corporation, College Station, TX). A significance threshold of *P*-values less than .05 was used for determining statistical significance.

## Results

Table 1 provides a comprehensive demographic overview of the study population.

**Table 1.** Characteristics of the study population, adults aged 58+, SHARE Switzerland, 2019/2020, *n* = 1645

	*n*	%
Sex		
Male	779	47.4
Female	866	52.6
Age groups (years)		
58–64	437	26.6
65–74	665	40.4
75+	543	33.0
Education		
Low	287	17.5
Middle	1022	62.1
High	336	20.4
Partnership status		
Has a partner	1233	74.9
No partner	412	25.1
Make ends meet		
Easily	903	54.9
Fairly easily	520	31.6
With difficulty	222	13.5
Language		
German	1159	70.5
French	427	25.9
Italian	59	3.6
Living area		
Urban	757	46.0
Rural	888	54.0
Self-rated health		
Poor/fair health	308	18.7
Good health	697	42.4
Very good/excellent health	640	38.9
ADL limitations		
No	1533	93.2
Yes	112	6.8
Mild cognitive impairment		
No	1535	93.3
Yes	110	6.7
Moderate physical activity		
No	138	8.4
Yes	1507	91.6
Vigorous physical activity		
No	660	40.1
Yes	985	59.9
Standardized health literacy score	Mean: 4.4 Min: 0	SD: 1 Max: 5.3

Number of observations for the whole sample.

The findings from probit regression analyses (Table 2 ), adjusted for all covariates, reveal that HL was positively associated with engagement in moderate and vigorous physical activity (AME: 0.02, *P* < .001; AME: 0.03, *P* < .01). Regarding MCI, the analysis showed mixed results. A higher HL score was generally associated with a decreased likelihood of MCI (AME: −0.01, *P* < .05) when including the HL score alone or with the variable assessing vigorous physical activity. The results were not statistically significant in the model that included both HL and moderate physical activity. However, engagement in moderate physical activity was associated with a decrease in the likelihood of MCI (AME: −0.06; *P* < .05). Similarly, vigorous physical activity was associated with a decrease in the likelihood of MCI (AME: −0.03; *P* < .01).

**Table 2.** Probit regression analysis of health literacy, physical activity levels, and mild cognitive impairment, accounting for the covariates, adults aged 58+, SHARE Switzerland, 2019/2020, *n* = 1645

	Moderate PA	Vigorous PA	MCI	MCI and mPA	MCI and vPA
Health literacy scores standardized	0.02***	0.03**	−0.01*	−0.01	−0.01*
	(0.01)	(0.01)	(0.01)	(0.01)	(0.01)
Moderate PA					
Yes				−0.06*	
				(0.03)	
Vigorous PA					
Yes					−0.03**
					(0.01)
Observations	1645	1645	1645	1645	1645

The table shows average marginal effects and standard errors in parentheses.

PA = physical activity. MCI = mild cognitive impairment. The first two columns show the results from probit regressions of physical activity levels on the standardized health literacy score and the covariates. The other three columns present probit regression analyses of MCI on the standardized health literacy score, with models that include no physical activity, moderate physical activity, or vigorous physical activity levels, as well as all covariates. The covariates include sex, age, education levels, partnership status, subjective financial situation, linguistic region, living area, self-rated health, and ADL limitations.

* *P* < .05,

** *P* < .01,

*** *P* < .001.

**Figure 2.**
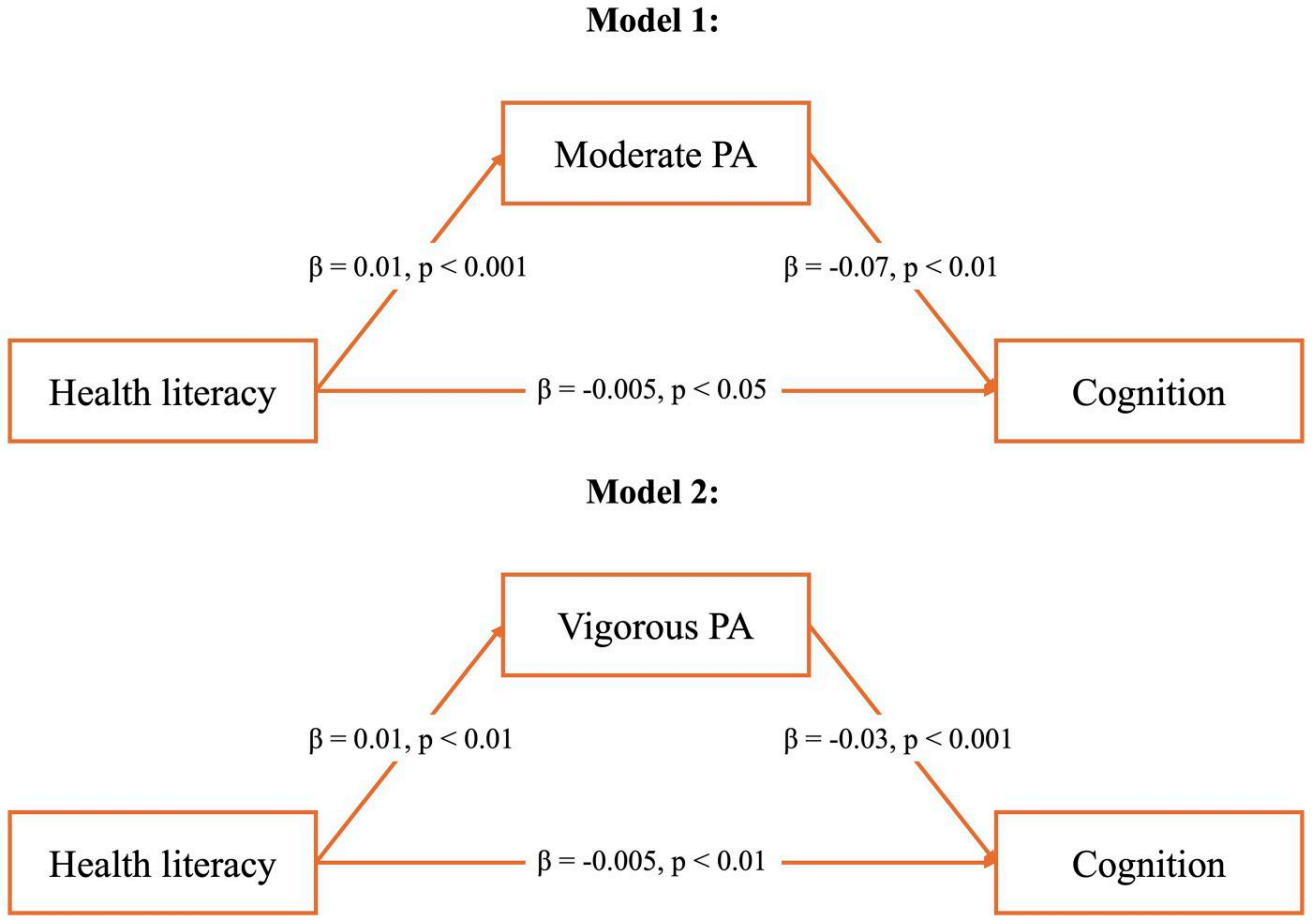
Analysis of the mediating role of physical activity levels in the association between health literacy levels and mild cognitive impairment, accounting for the covariates, adults aged 58+, SHARE Switzerland, 2019/2020, *n* = 1645. PA = physical activity. The figure shows the results from two structural equation modeling (SEM) analyses; the first model investigates the mediating role of moderate physical activity in the association between health literacy levels and mild cognitive impairment with the covariates. The second model shows the mediation analysis of vigorous physical activity in the association between health literacy levels and mild cognitive impairment with the covariates. The covariates include sex, age, education levels, partnership status, subjective financial situation, linguistic region, living area, self-rated health, and ADL limitations.

Figure 2 shows the results from the two SEM analyses that assess the mediating role of physical activity in the relationship between HL levels and MCI. The results from the first model exploring the mediating role of moderate physical activity show that HL is positively associated with physical activity (β = 0.01, *P* < .001), moderate physical activity is negatively associated with MCI (β = −0.07, *P* < .01) and HL is negatively associated with MCI (β = −0.005, *P* < .05). In addition, about 12.9% of the association of HL on the likelihood of MCI is mediated by engagement in moderate physical activity. The second model examines the mediating role of vigorous physical activity. The results show that HL is positively associated with vigorous physical activity (β = 0.01, *P* < .01), vigorous physical activity is negatively associated with cognition (β = −0.03, *P* < .001) and HL also shows a direct negative association with cognition (β = −0.005, *P* < .01). Moreover, about 6.7% of the association of HL on the likelihood of MCI is mediated by vigorous physical activity levels among the study population. Each of the two models demonstrated excellent fit indices, with all models achieving a CFI and TLI of 1.000 and an RMSEA of 0.000.

## Discussion

Drawing on data from a comprehensive population-based survey of 1645 adults aged 58 and older, the study indicates that higher HL is significantly associated with greater engagement in both moderate and vigorous physical activity, which, in turn, correlates with a reduced likelihood of having MCI. Notably, moderate physical activity mediates 12.9% and vigorous physical activity 6.7% of the relationship between HL and MCI, underscoring the importance of physical activity as a protective factor against cognitive decline. The study suggests that promoting both HL and physical activity could be promising strategies to mitigate the risk of cognitive decline.

### The role of health literacy in enhancing physical activity to prevent or delay cognitive impairment

The present findings align with previous research suggesting that HL can enhance knowledge, motivation, and self-efficacy with regard to healthy behaviors, including starting and sustaining physical activity which may contribute to maintaining cognitive function in later life [28, 29]. A longitudinal study using data from the English Longitudinal Study of Ageing found that a combination of higher HL (defined as answering all four questions on an HL assessment correctly) and good cognitive functioning (measured by higher memory and verbal fluency scores) are associated with consistent participation in moderate to vigorous physical activity over 8 years, demonstrating the interplay between cognitive function, HL, and physical activity in later life [30]. In addition to the mediation effects observed from the present study, our results support theories positing that lifestyle factors play a crucial role in the HL–cognitive function association, potentially offering actionable targets for public health interventions [7]. Different research discussing the health consequences of physical inactivity in older adults indicated that physical inactivity is associated with higher risks of all-cause mortality and various diseases, including cardiovascular diseases and certain cancers, a decline in functional and cognitive abilities, higher prevalence of mental health issues, such as depression and anxiety and higher burden on healthcare systems [31, 32]. Thus, these insights underscore the vital importance of promoting HL as a means to enhance overall well-being and cognitive health in the aging population.

### Potential reverse causation

Current evidence on the association between HL and cognition suggests that higher levels of HL are associated with better cognitive functioning. However, it is important to consider that the relationship might also exhibit reverse causation or be bidirectional. This means that while HL can influence cognitive function, cognitive function could also influence an individual’s HL. For instance, a study showed that accounting for cognitive abilities significantly weakens the link between poor HL and worse physical health, highlighting the potential crucial mediating role of cognition [33]. In addition, the results from another study indicated that cognitive decline significantly influences HL among older adults without dementia, besides, more rapid declines in executive function and episodic memory are associated with lower HL levels over time [34]. Additional studies found that cognitive decline not only impairs general cognitive functions but specifically affects the ability to process and understand health information, which is crucial for making informed decisions in older age [35, 36]. Specifically, higher cognitive abilities, both fluid (related to processing new information) and crystallized (related to accumulated knowledge), significantly improved the ability to perform health-related tasks, suggesting that cognitive abilities play a critical role in how well older adults manage health-related information and tasks [37]. Therefore, while the predominant direction of association suggests that HL positively impacts cognitive functioning, the relationship can be complex and potentially moderated by multiple factors, including age, education, and overall health status. Additionally, we acknowledge that using cross-sectional data for mediation analysis poses limitations in establishing a causal sequence. This design constrains our ability to confirm whether physical activity mediates the relationship between HL and cognitive functioning, as longitudinal data would be necessary to confirm the causal ordering of these variables. Consequently, any mediation effects observed in the study should be considered tentative and exploratory.

### Practical implications and future research

The findings of this study have several important practical implications for improving HL and promoting physical activity among older adults, both critical for maintaining cognitive function as they age. First, there is a need to develop and implement targeted HL programs [38]. These should focus on equipping older adults with the necessary skills to effectively access, understand, and utilize health information [38]. Additionally, it is essential for healthcare providers to routinely incorporate physical activity discussions and prescriptions into standard care for older adults [39]. Community-based physical activity programs that are accessible and tailored to varying levels of mobility and health status can facilitate this [39]. Policy initiatives should also support environments conducive to physical activity, such as creating safe walking paths and senior-friendly sports facilities [40]. Furthermore, there should be a concerted effort to utilize media and community outreach to promote the dual benefits of HL and physical activity in preventing cognitive decline, including workshops and seminars that teach manageable cognitive and physical exercises for home practice.

Future research can further elucidate the connections between HL, physical activity, and cognitive function. Longitudinal studies are needed to assess the causal relationships between these variables over time, which would help to understand the long-term effects of HL improvements on lifestyle changes and cognitive health. Additionally, exploring other lifestyle factors, such as smoking and diet could offer a more comprehensive view of the relationship between HL and cognitive function. Intervention studies could be invaluable in testing the effectiveness of programs designed to enhance HL and in examining how these improvements influence physical activity levels and cognitive outcomes. Finally, the role of technology in improving HL and promoting physical activity should not be overlooked. The potential of apps, wearable devices, and other technological innovations could be significant in tracking activity levels and providing educational content tailored to the needs of older adults.

### Limitations

Our study acknowledges several limitations. Firstly, relying on self-reported data for measuring physical activity could introduce response biases, as participants might overestimate their activity levels or misunderstand the activity intensity definitions provided in the survey. The same concerns apply to the use of the self-rated measures of HL. Additionally, the cross-sectional design of our study limits the ability to infer causality between HL, physical activity, and cognitive function. As the main SHARE questionnaire lacks a consistent HL measure across waves, we used data from the Swiss paper-and-pencil questionnaire, which included this measure only once in Wave 8. This limitation restricts our analysis to a single wave and underscores the need for future waves to incorporate HL measures. Moreover, although attrition and nonresponses in the SHARE study could skew results, potentially not including the most vulnerable individuals, the high response rate and the absence of critical tendency among excluded participants support our conclusions. Addressing these limitations in future studies could involve incorporating more objective measures of physical activity, such as wearable devices, and employing longitudinal designs to track changes over time.

## Conclusion

In conclusion, our study highlights the potential of HL as a modifiable factor that could be targeted to promote physical activity, which can, in turn, be a lever to prevent cognitive impairment in older adults. Higher HL levels improve individuals’ knowledge and understanding of health information, which can significantly contribute to lifestyle choices such as engaging in physical activity that protects cognitive health. Therefore, targeted strategies that improve HL could be pivotal in preventive health measures aimed at combating cognitive decline. This study lays the groundwork for future research to explore these associations more deeply through longitudinal designs that can better ascertain causality and track changes over time. Ultimately, by continuing to investigate these critical relationships, we can better tailor interventions to meet the needs of the aging population, ensuring that older adults lead not only longer but healthier lives cognitively.

## Supplementary Material

ckae209_Supplementary_Data

## Data Availability

This paper uses data from SHARE Wave 8 (https://doi.org/10.6103/SHARE.w8.800; see Börsch-Supan [20]). Study data already de-identified are available to the scientific community upon submitting a data requestion application to the SHARE study.
