# Supplementary material for: Physical activity partially mediates the association between health literacy and mild cognitive impairment in older adults: cross-sectional evidence from Switzerland
Source: Eur J Public Health. 2025 Jan 3;35(1):134–40. doi: 10.1093/eurpub/ckae209 (PMC11832147; doi:10.1093/eurpub/ckae209)
Supplement: ckae209_Supplementary_Data [file ckae209_supplementary_data.docx]

**Appendices**

**Appendix 1: Immediate and delayed memory recall**

**Question 1:** Now, I am going to read a list of words from my computer screen. We have purposely made the list long so it will be difficult for anyone to recall all the words. Most people recall just a few. Please listen carefully, as the set of words cannot be repeated. When I have finished, I will ask you to recall aloud as many of the words as you can, in any order. Is this clear? Now please tell me all the words you can recall.

Answer: list of the words the respondent correctly recalls.

**Question 2:** A little while ago, I read you a list of words and you repeated the ones you could remember. Please tell me any of the words that you can remember now?

Answer: list of the words the respondent correctly recalls.

*List 1: Hotel, River, Tree, Skin, Gold, Market, Paper, Child, King, Book*

*List 2: Sky, Ocean, Flag, Dollar, Wife, Machine, Home, Earth, College, Butter*

*List 3: Woman, Rock, Blood, Corner, Shoes, Letter, Girl, House, Valley, Engine*

*List 4: Water, Church, Doctor, Palace, Fire, Garden, Sea, Village, Baby, Table*

**Appendix 2: Verbal fluency**

**Question 1:** Now I would like you to name as many different animals as you can think of. You have one minute to do this.

Answer: The score is the sum of acceptable animals. Any member of the animal kingdom, real or mythical is scored correct, except repetitions and proper nouns. Specifically, each of the following gets credit: a species name and any accompanying breeds within the species; male, female and infant names within the species.

**Appendix 3: List of items from the European Health Literacy Survey questionnaire (HLS-EU-Q16)**

**Question 1:** First, we would like to ask you how comfortable you feel when dealing with health-related information. For you, how easy or difficult is it to…

Answer categories: "Very easy", "Fairly easy", "Fairly difficult", "Very difficult.”

1. Find information on treatments of illnesses that concern you?
2. Find out where to get professional help when you are ill?
3. Understand what your doctor says to you?
4. Understand your doctor or pharmacist’s instruction on how to take prescribed medicine?
5. Use the information the doctor gives you to make decisions about your illness?
6. Judge when you may need to get a second opinion from another doctor?
7. Follow instructions from your doctor or pharmacist?
8. Find information on how to manage mental health problems like stress or depression?
9. Understand health warnings about behaviour such as smoking, low physical activity, and excessive drinking?
10. Understand why you need health screenings?
11. Judge if the information on health risks in the media is reliable?
12. Decide how you can protect yourself from illness based on information in the media?
13. Understand information in the media on how to be healthier?
14. Find out about activities that benefit your mental well- being?
15. Understand advice on health from family members or friends?
16. Judge which everyday behaviour is related to your health?

**Appendix 4: Physical activities**

**Question 1:** We would like to know about the type and amount of physical activity you do in your daily life. How often do you engage in vigorous physical activity, such as sports, heavy housework, or a job that involves physical labour?

Answer categories: “More than once a week”, “Once a week”, “One to three times a month”, Hardly ever or never.”

**Question 2:** How often do you engage in activities that require a moderate level of energy such as gardening, cleaning the car, or doing a walk?

Answer categories: “More than once a week”, “Once a week”, “One to three times a month”, Hardly ever or never.”
